# Novel Insights Into Mesothelioma Therapy: Emerging Avenues and Future Prospects

**DOI:** 10.3389/fonc.2022.916839

**Published:** 2022-06-17

**Authors:** Lukasz Kuryk, Giulia Rodella, Monika Staniszewska, Katarzyna Wanda Pancer, Magdalena Wieczorek, Stefano Salmaso, Paolo Caliceti, Mariangela Garofalo

**Affiliations:** ^1^Department of Virology, National Institute of Public Health National Institute of Hygiene (NIH)—National Institute of Research, Warsaw, Poland; ^2^Department of Pharmaceutical and Pharmacological Sciences, University of Padova, Padova, Italy; ^3^Centre for Advanced Materials and Technologies, Warsaw University of Technology, Warsaw, Poland

**Keywords:** mesothelioma, oncolytic adenoviruses, immunotherapy, immune checkpoint inhibitors, combination therapy

## Abstract

Malignant mesothelioma is a rare and aggressive cancer that develops in the thin layer surrounding the mesothelium and is mainly caused by asbestos exposure. Despite improvements in patient prognosis with conventional cancer treatments, such as surgery, chemotherapy, and radiotherapy, there are still no curative treatment modalities for advanced disease. In recent years, new therapeutic avenues have been explored. Improved understanding of the mechanisms underlying the dynamic tumor interaction with the immune system has led to the development of immunotherapeutic approaches. Numerous recent clinical trials have shown a desire to develop more effective treatments that can be used to fight against the disease. Immune checkpoint inhibitors, oncolytic adenoviruses, and their combination represent a promising strategy that can be used to synergistically overcome immunosuppression in the mesothelioma tumor microenvironment. This review provides a synthesized overview of the current state of knowledge on new therapeutic options for mesothelioma with a focus on the results of clinical trials conducted in the field.

## Introduction

Malignant pleural mesothelioma (MPM) is an aggressive and rare type of cancer that develops within the thin layer surrounding the pleura made of mesothelial cells (1, 2). It is considered an occupational disease because of its close association with previous asbestos exposure in the workplace. In fact, males working in places with high quantities of this harmful agent are more commonly affected than women, who, generally, have lower exposure to asbestos (3). The worldwide incidence of this cancer has risen steadily over the last decade, and an increase in the number of cases in the incoming period is predicted. The main reasons for this are the long latency period between the first exposure and the disease’s appearance and the extensive use of asbestos during the 1970s in many industrial sectors (4). Despite current awareness of its hazardous nature, asbestos is still used widely in industrialized countries such as India, Russia, and Brazil; thus, this problem is ongoing (1). Moreover, exposure during the process of removing this material from buildings and incidental exposure are serious concerns that will remain until the material is completely banned worldwide (3). Sadly, the malignancy is almost universally lethal, and the median survival time is between 9 and 12 months from diagnosis (3). The low chance of healing is mainly correlated with the long period for which the tumor remains dormant (up to 50 years), so when its existence becomes apparent, it is too late to intervene and remove the widespread malignancy (1, 2, 5). Some treatments are currently available, although they are not resolutive, and intense research is needed to provide hope for mesothelioma patients (6).

Herein, we focus on the current standards of care and future prospects for mesothelioma therapy. Current clinical study data with immunotherapies are bringing some light to this area, and vast improvements in mesothelioma therapy may arise in forthcoming years. Therefore, in this review, we highlight the clinical prospects of the use of oncolytic viruses in combination with immune checkpoint inhibitors (ICIs). We hope that this review will support the global community in the design of appropriate immune interventions for the treatment of mesothelioma.

## Pathogenesis

MPM results from the neoplastic transformation of mesothelial cells that form a thin single layer that covers the entire surface of the pleura. This malignancy is mainly caused by asbestos inhalation, since about 80% of cases are associated with a prior exposure (7). Asbestos, a dangerous silicate mineral, is found naturally in rocks and soils and can be classified into two categories according to shape: the curly type consisting of serpentine fibers (called chrysotile) and the amphibole type with a needle-like form. The latter is further subdivided into crocidolite, amosite, anthophyllite, tremolite, and actinolite (8). The ability of this mineral to cause disease is highly associated with the thickness and length of the fibers as well as its heaviness and the length of exposure. For instance, the correlation between amphibole fiber exposure and MPM induction is well established. The long and thin shape of the fibers allows them to penetrate deeply into the lungs. Crocidolite is considered the most carcinogenic form of asbestos (7). However, the etiology of the neoplasm is not completely understood, and it might not be related only to asbestos but also to other concomitant factors (9). New insights into other possible trigger factors are under investigation, such as exposure to minerals with features similar to those of asbestos that may act in the same way, and radiation from therapy or workplace exposure, which is well-known for its carcinogenic potential. Furthermore, an oncogenic virus named simian virus 40 seems to play a role in mesothelioma development, although the findings on this virus require further validation (9). Several studies showed that the virus it is able to express the large T antigen (Tag) which activates pathways related to cell growth and prolonged survival (10). Among them, Phosphatidylinositol 3-kinase (PI3K/Akt) is well known to be involved in the blockage of apoptosis by cell cycle dysregulation and suppression of pro-apoptotic proteins, and it has been highlighted as the most critical mechanism responsible for malignant cells evolution (11). According to this, *Cacciotti et al.* demonstrated that SV40-dependent growth factors (i.e. VEGF) are released in presence of asbestos fibers, and they trigger Akt phosphorylation on mesothelial cells, thus leading to progressive resistance to apoptosis (10). This evidence may explain the potential cocarcinogenic property of SV40 virus when combined to toxic agents’ exposure. Therefore, chronic exposure to asbestos remains the main cause of MPM, and the asbestos’ fiber shape and length-to-width ratio determine whether the particles can penetrate the lung epithelium to reach the pleural space (3). Once there, they can exert harmful activity through a combination of different pathways. First of all, the fibers cause pleural irritation and a continuous cycle of scratching, damage, and repair. This condition of prolonged chronic inflammation leads to scarring or tumor progression in the mesothelial cells (3). Another major consequence of asbestos exposure is the generation of reactive oxygen species (ROS), known for their toxic nature and capacity to induce DNA damage (3). The production of these dangerous free radicals derives from both the iron content of the asbestos fibers themselves and from phagocytosis by macrophages (12) these scavenger cells are not able to process them, consequently leading to the excessive release of ROS (7). First, the mineral origin of asbestos explained the presence of iron ions which can catalyzed reactions generating oxygen and nitrogen radical residues (ROS and RNS). Secondly, alveolar and peritoneal macrophages can phagocyte these harmful fibers thus leading to inflammation with both production of ROS/RNS and cytokines (TNFa, IL-6) (13). Collectively, the accumulation of these reactive chemical entities (O_2_·^−^, ·OH, and H_2_O_2_) act as second messengers to trigger cell pathways responsible for uncontrolled proliferation as MAPK, PI3K/Akt and NF-kB cascades, and DNA damage (14). Remarkably, ROS and RNS, together with asbestos fibers themselves, might induce mutagenicity and genotoxicity in mesothelial cells through physical interaction with the mitotic system and indirect damage of DNA and chromosome, respectively (13). Despite the protective role of the intrinsic antioxidant system of manganese superoxide dismutase (Mn SOD) and catalase (CAT), asbestos-generated reactive species cause several DNA injuries, like DNA single-strand breaks (SSB), chromosome fragments and 8-hydroxydeoxyguanosine (8-OHdG); this latter is the main product of oxidative damage which induces G→T and A→C transversions. These genomic substitutions are involved in spontaneous oncogene expression and subsequent malignant evolution of cells (12). Moreover, engulfed asbestos fibers can hamper the process of mitosis through their ability to pierce the mitotic spindle. This results in mutations, aberrant chromosomal structure, and the aneuploidy of mesothelial cells (7). Several DNA repair mechanisms attempt to resolve this disruption of the genome, but if repair enzymes are defective, they could establish another pathway that promotes tumor growth and could act synergistically with other pathogenic processes (15). Moreover, such fibers are capable of binding to crucial cellular proteins, making them unable to exert their specific roles, therefore weakening mesothelial cell function (7). Both asbestos-exposed cells and macrophages can release high amounts of inflammatory cytokines and growth factors, such as tumor growth factor β and vascular endothelial growth factor (VEGF), which generates a milieu propitious for tumor outgrowth (7). As previously mentioned, these tumor growth factors can activate the PI3K/Akt pathway which has been shown to play a critical role in tumor development and evolution (10). To be more precise, the anti-apoptotic activity could be identified into the main downstream Akt target: mTOR (16). In three-dimensional cultures and ex vivo, mTOR has been observed as the central regulator responsible for the apoptotic resistance of mesothelial cells through the activation of oncogenic genes, like S6K (17). Interestingly, it has been observed that the miRNAs can work as both tumor suppressor or oncogenes by targeting specific pathways implicated in carcinogenesis. A recent study evaluated miRNAs expression in MPM and identified their downregulation as subsequent inhibition of several cascades (like PIK3-Akt), meaning that it could be addressed to find potential new pharmacological targets (18). Alveolar macrophages can also secrete inflammatory cytokines, as TNFalfa, which is implicated in cell apoptosis and compensatory proliferation due to the binding of TNFR or through cooperative interaction with oxidants to activate MAPK/NF-κB pathway (19). Therefore, the asbestos-induced mitogen-activated protein kinase (MAPK) activation is another interesting pathogenic mechanism under investigation. The MAPKs are a family of enzymes involved in the regulation of a variety of signaling activities associated with the extracellular environment, including the phosphorylation of proteins and transcription factors (20). In addition, they can participate in key cellular events such as apoptosis, proliferation, and differentiation (20). Asbestos fibers appear to be able to trigger the activity of a member of the MAPK family, extracellular-regulated kinase (ERK), through interaction with epidermal growth factor receptors (EGFRs) that are located on the cell membrane (21). In fact, these receptors can compete with the physiological ligand (EGF) for the receptor binding site; fiber engagement thereby induces EGFR dimerization and consequent activation, which leads to ERK phosphorylation (21). When ERKs are activated, they translocate to the nucleus, where an intracellular cascade is triggered, finally inducing protooncogene activation. This means that some factors involved in cell cycle progression, such as c-*fos* and c-*jun*, are then overexpressed, which might stimulate mesothelial cell carcinogenesis (21). EGFR and, consequently, ERK can be stimulated *via* the generation of ROS, either by asbestos fibers directly or by cells engulfing them (20). On top of that, NF-kB-dependent genes can be activated by asbestos fibers, thus driving to other proto-oncogene activation, like c-myc. Since NF-kB activation is involved in inflammatory pathways, it is conceivable to correlate fibers exposure and consequent tumor evolution (19). In addition to these mechanisms that are likely to be related to asbestos exposure, some people seem to develop the disease despite having no contact with the dangerous mineral. This intriguing fact may be explained through a predisposition to MPM dictated by a particular genomic mutation of the BRCA1-associated protein-1 (BAP1) gene (4). This encodes a hydrolase enzyme localized within the nucleus, and it can regulate genes involved in cell cycle progression, DNA repair, and cell differentiation. Interestingly, germline mutations of BAP1 have been highlighted in families with a high incidence of mesothelioma, suggesting a possible implication of this genetic change in tumor evolution. A study was conducted to shed light on the prevalence and clinical predictors of germline cancer susceptibility (22). They found that 12% of considered MM patients carry genomic mutations not correlated to asbestos exposure, and other 13 genes (apart from BAP1) have been identified as predisposing factors linked to MM development, including *TMEM127, CHEK2, MRE11A, VHL, WT1*, and *SDHA* (22). These data suggest the possibility of prevention and early detection of the disease through a genetic screening of these biomarkers potentially predisposing tumor development. In addition to that, cytogenetic tests measuring chromosome aberrations in peripheral blood lymphocytes (PBL) has been demonstrated to be a reliable biomarker to detect cancer risk, independent from toxic agents’ exposure (23). Among them, the evaluation of micronuclei in PBL in MM patients has shown (24) a higher frequency of micronuclei compared to lung cancer and the healthy counterparts. Collected evidence permitted to speculate that several genetic predisposing factors might be correlated with malignant evolution of mesothelial cells. As soon as mesothelioma develops, different categories of cell type can be identified according to morphological shape: epithelioid, sarcomatoid, and biphasic (7). The epithelial subtypes are cuboidal and those with more uniform histological features. While the sarcomatoid type is characterized by spindle-like cancer cells, the biphasic type comprises both subtypes, showing mutual characteristics from each of them (3). The sarcomatoid type is considered the most aggressive kind of MPM. It has a poor prognosis and is associated with a shorter survival time (25). The first clinical features of the disease are breathlessness and chest pain due to the localization of the primary tumor within the pleural space. Furthermore, unexplained pleural effusion might occur, and this is usually how patients discover their problem (3). With the progression of the malignancy, pain is often combined with fatigue, weight loss, fever, and cachexia (3). Despite the aggressiveness of MPM, metastases rarely occur and are usually detected post-mortem (3).

Interestingly, it has been observed that the miRNAs can work as both tumor suppressor or oncogenes by targeting specific pathways implicated in carcinogenesis. A recent study evaluated miRNAs expression in MPM and identified their downregulation as subsequent inhibition of several cascades (like PIK3-Akt), meaning that it could be addressed to find potential new pharmacological targets (18).

## Current Standards of Care for Mesothelioma Treatment

The prognosis of MPM depends on several benchmarks such as tumor extension, differentiation, and histological subtype. The worst prognosis is associated with cases with a high white blood cell count, chest wall pain, and the sarcomatoid cell type (3). The management of this disease comprises many approaches designed to increase the survival prospects of affected patients (1). The chances of a full recovery are limited, probably because the disease tends to be discovered at the advanced stage; for this reason, the available treatments are often considered non-resolutive and are only able to partially improve the lifestyles of patients (6). Next, we discuss the available conventional treatments.

### Surgery

Surgical resection is considered an option for mesothelioma treatment, although its effectiveness is highly debatable. The main problem is that the tumor surrounds all the pleural surfaces as well as the interlobular fissures, so an invasive operation is required to remove the affected tissue (26). Less radical approaches, such as a debulking operation, or more invasive and potentially curative approaches, such as extrapleural pneumonectomy (EPP), can be used (27). The former approach can be performed through video-assisted thoracic surgery or thoracotomy with the maximum elimination of the tumor burden achievable without removing the underlying lung, pericardium, or diaphragm (26). The primary advantages of the operation are the reductions in fluid recurrence and pleural effusions, which are subsequently correlated with increased survival. Although these factors could be promising, their effectiveness has not been confirmed by randomized trials (26). On the other hand, EPP is the most radical procedure and has the goal of removing all the gross tumor through the resection of the parietal and visceral pleura, ipsilateral lung, pericardium, and diaphragm (28). Unfortunately, only a minority of patients are eligible for EPP, owing to late diagnosis of the disease and the consequent involvement of the lymph nodes and impaired cardiopulmonary function (29). Moreover, only patients presenting with the epithelioid subtype should be considered suitable for treatment with surgical methods (29), since this treatment option can be considered either curative or fatal. The operative mortality is around 6%, but the median survival improvement is more than 2 years with better management of the thoracic issues (3). The MARS pilot trial (ISRCTN95583524), in which 50 patients were randomized to receive chemotherapy, EPP, and hemithoracic radiotherapy, showed a mortality rate of 18% in the EPP group and a better survival rate in the non-EPP group (30). In summary, the outcomes from this clinical trial did not demonstrate a benefit from EPP over chemotherapy alone in terms of survival and quality of life (30). Improved outcomes were reported with the combination of adjuvant therapy and local radiotherapy in a multi-modal regimen; this research, though limited, speculated that radical surgery in the form of EPP within trimodal therapy provides no benefit and possibly harms patients (26, 30).

### Chemotherapy

Considering that not all types of mesothelioma are resectable, different management options need to be available (3). Although there is no curative treatment, polychemotherapy is recommended as a front-line therapeutic regimen for nonresectable MPM (29). This standard of care consists of the combination of pemetrexed and cisplatin, instead of using the platinum compound alone (31). The former is an inhibitor of different proteins, including dihydrofolate reductase and thymidylate synthase, which are both involved in DNA synthesis; cisplatin, on the other hand, is a DNA-intercalator that prevents DNA replication within cancer cells (3). A multicenter phase III study including 456 patients demonstrated an improvement in overall survival of nearly 3 months following treatment with the combination therapy compared to treatment with cisplatin alone (32). This evidence led to the FDA approval of this type of treatment for extending life expectancy and helping with the disease management of many mesothelioma patients (26). Moreover, two studies have suggested carboplatin as an attractive alternative to cisplatin in the elderly and unfit population; the main reason for this is the lower symptomatic toxicity and the easier administration, without the need for prolonged hydration with intravenous fluids (33, 34). In light of these randomized trials, pemetrexed used in combination with cisplatin or carboplatin is considered the first choice for treatment, because it has been demonstrated to have lower toxicity and can be administered on an easier schedule (3-weekly outpatient injections) (Pemetrexed, 500 mg/m^2^, in 100 mL of normal saline for 10 minutes followed by the administration of cisplatin 30 minutes later at a dose of 75 mg/m^2^ over 2 hours) compared to other therapies such as doxorubicin, methotrexate, 5-fluorouracil, and cisplatin alone (26). However, some patients do not benefit from the potential advantages of this kind of therapy; therefore, other approaches are needed to provide supportive care to non-responders. One example is the use of cisplatin in combination with gemcitabine, a false nucleotide that, once incorporated into the DNA, does not allow the synthesis of the genome (3). Nevertheless, this type of therapy has palliative effects. The synergistic combination of these two drugs showed an objective response rate of 33% in a multicenter phase II study as well as benefits in terms of symptom management and quality of life (31). Another novel strategy is represented by the combination of standards of care (cisplatin and pemetrexed) with the antiangiogenic drug bevacizumab. A large phase III trial (NCT00651456) including 448 patients showed that the combinatory treatment induced a longer survival than the standard of care alone (35) thus in light of these evidences, it should be considered as a suitable treatment. Interestingly some US and French guidelines have validated this new approach to treat unresectable MPM patients (36) even if, it has not received the FDA and EMA approval yet, nevertheless this preliminary evaluation appeared to be encouraging. Other alternatives under investigation are imatinib and gefitinib; however, to date, studies examining their effects have not produced convincing evidence to support their approval (27).

### Radiotherapy

Malignant pleural mesothelioma is usually resistant to traditional radiotherapy, and due to its toxic effects on the lungs and adjacent organs, its use is not indicated either (26). A retrospective study involving patients treated with hemithoracic radiation after surgery showed a median survival time of 13.5 months but also severe pulmonary toxicity in 10% of cases (37). For this reason, only intensity-modulated radiotherapy is suggested for the treatment of relatively small volumes of tumor using short schedules (1). The purpose is palliative, to reduce pain and provide relief from the chest wall infiltration (27). Furthermore, radiotherapy is considered a prophylactic treatment due to the risk of seeding in the scar that could appear after invasive diagnostic techniques and surgery, which could then trigger the outgrowth of a subcutaneous tumor (26). In fact, a randomized study showed that, upon treating scars with three sessions of early local radiation, this risk reduced from 40 to 0% (38), thus suggesting that this approach might improve the effectiveness of combined treatments. However, due to the lack of clinical studies with definitive results, the use of radiotherapy remains within the settings of clinical trials and palliation.

### Multimodal Therapy

Due to the low effectiveness of monotherapies, multimodal treatment involving the combination of three therapeutic modalities—surgery, chemotherapy, and radiotherapy—is used (39). A study performed by Rea et al. showed that administering chemotherapy through four cycles of carboplatin/gemcitabine, followed by the use of EPP and postoperative radiotherapy, was associated with a median overall survival rate of 25.5 months (40). This evidence suggests the feasibility and partial effectiveness (40) of the treatment option. A comparable study was also set up by the European Organization for Research and Treatment of Cancer (EORTC) involving 59 patients with MPM, where 37 received a trimodal treatment consisting of cisplatin/pemetrexed, EPP, and final postoperative radiation (39). The results suggested the suitability of this approach for selected patients with early-stage disease and in specialized centers (39). Despite the promising evidence, another study highlighted the low usefulness of hemithoracic radiotherapy after neoadjuvant chemotherapy and surgery. In fact, this approach does not seem to provide improvements in terms of either survival or quality of life, and, surprisingly, radiation was associated with increased adverse effects (41). Other trials are currently ongoing to further investigate the effect of this approach, such as MARS 2 (NCT02040272), which is analyzing the outcomes of platinum-based therapy and pleurectomy/decortication (P/D) versus chemotherapy alone (42). Intriguingly, multimodal treatment has also involved other innovative strategies such as immunotherapy (NCT02707666), photodynamic treatment (NCT02662504), and immunogene therapy (NCT01119664) in the search for new and more effective synergistic combinations (42).

### Local Therapies

Few strategies for local therapeutic delivery have been developed and tested over the past years in mesothelioma treatment. The major rationale of these therapies is: (i) to diminish the risk of therapeutic agent inactivation before reaching the target tumour mass; (ii) to elevate the concentration of drugs in the tumour microenvironment (iii) to reduce toxicity (43). Development of nanotechnology in biomedicine opens many opportunities in cancer treatment. In oncology the preferentially route of nanoparticles (NPs) administration is systemic injection. However, the best approach to administer therapies in malignant pleural mesothelioma seems to be the local treatment e.g. pleural injection or inhalation (43). The most tested NPs for MPM treatment are liposomes. Liposomes can deliver both hydrophobic and hydrophilic agents, but also genetic material. In malignant pleural mesothelioma treatment, liposomes are used to deliver chemotherapeutics, such as doxorubicin (44, 45), or pemetrexed (46, 47), with the main objective being to enhance the concentration of active drugs in the peritoneal cavity and to extend the release of the compounds. Ando et al. showed that administration into the pleural cavity of pemetrexed inside liposomes resulted in more profound suppressive effect on tumour growth in mesothelioma mouse model, compared to pemetrexed alone (43). Other researchers investigated (48, 49) liposomal accumulation in mesothelioma tumour bearing mice and in healthy animals. The study reported that small liposomes or PEGylated liposomes had higher retention. However, the clearance of liposomes system from the pleural cavity was the same comparing normal mice to tumour bearing ones (49). Another NPs also have been tested in malignant pleural mesothelioma therapy such as gold NPs (50) or pH-responsive polymeric NPs (51, 52). Promising findings have been reported by Schulz et al. The authors showed that only nano-formulated paclitaxel can significantly increase animal survival compared to those mice treated only with surgery or with the supplementation of free paclitaxel. Enhanced efficacy was related because NPs extended release of chemotherapeutics in the intraperitoneal cavity.

Tumour Treating Fields (TT Fields) are low intensity (1–3 V/cm root-mean-square (RMS)), intermediate frequency (100–500 kHz) with antimitotic effects on cancerous cells (53, 54). TT Fields are given locally at the tumour area. The efficacy of TT Fields for the treatment of unresectable malignant pleural mesothelioma in combination with pemetrexed plus cisplatin or carboplatin was assessed in Phase 2 STELLAR clinical trial (55). Based on the STELLAR study, TT Fields in combination with pemetrexed and platinum-based chemotherapeutics were approved by the FDA and received a CE mark in Europe as first-line therapy for unresectable, locally advanced, or metastatic malignant pleural mesothelioma (56). TT Fields at a frequency of 150 kHz exhibited the highest cytotoxicity to mesothelioma cells, led to an enhanced number of DNA double-strand breaks, and diminished expression of Fanconi Anemia (FA)-BRCA DNA repair pathway proteins. The co-treatment of TT Fields with cisplatin or pemetrexed significantly enhanced treatment efficacy (56, 57). Nevertheless, there are a few limitations of the STELLAR study that should be mentioned. The study was a prospective, single-arm, non-randomized, open-label phase II trial, designed to study the safety and efficacy of NovoTTF-100L simultaneously with pemetrexed and cisplatin or carboplatin in mesothelioma patients (NCT02397928). Therefore, reported results should be interpreted with precautions as the study is lacking control arm.

### Immune Checkpoint Inhibitors

The immune system plays a crucial role in controlling MPM development and outgrowth, as is shown by the persistent chronic inflammation and local immunosuppressive milieu (6). In fact, it has been demonstrated that a greater intratumoral infiltration of cytotoxic CD8+ T cells corresponds with enhanced survival (1). Moreover, the few reported cases of tumor regression could have been caused by a direct immune reaction aimed at removing all malignant cells. In the past years, it has been demonstrated the ability of mesothelioma clones to act as antigen presenting cells when exposed to common recall antigens; through the assessment of ICAM1, MHC class II and B71 presence, it was clear their potential capability to present antigens responsible for CD4 T cells response. This observed immunogenicity may elicit an adaptive immune response towards the tumor as proved in immune-induced regression (58). In parallel, mesothelioma cells can block Th-1 reaction upon the stimulation of TGF-β-mediated pathway (59). Meaning that both costimulatory and immunosuppressive features are carried by this varied tumor environment, rendering the development of treatment approaches more challenging. Nevertheless, another correlation has been found in a cohort study: the higher the PD-L1 expression, the worse the prognosis. It has been demonstrated that it is an independent prognostic factor in MPM, responsible for significant survival disadvantage (60). This is due to the capacity of this ligand to bind to the corresponding receptors exposed on T cells and subsequently trigger inhibitory signaling. Thereby, the functionality of the immune system is limited, resulting in faster disease progression (1). Therefore, among the immunotherapeutic strategies available, ICIs have been considered potential candidates for mesothelioma treatment. Their purpose is to block the immune checkpoints that are overexpressed in the tumor microenvironment so that immune cell activity can be restored (1). Many clinical trials are now investigating the effect of this innovative therapy at different stages of malignancy and in combination with other standards of care (**Table 1**). In addition to that, they are examining potential biomarkers to predict outcomes following immunotherapeutic treatment; the most thoroughly studied is PD-L1 expression, which has been highlighted as an important predictive factor but not exclusive. Indeed, PD-L1 expression might be heterogeneous among different patients as well as varied over the time, leading to wrong interpretations (61). Therefore, its usage can be combined with other additional biomarkers to achieve a better comprehension of clinical effectiveness. Specifically, ICIs have been studied as a neoadjuvant therapy, first-line treatment, or second-/third-line treatment, as shown in **Table 1**.

**Table 1:** Clinical trials using immune checkpoint inhibitors.

IMMUNE CHECKPOINT INHIBITORS		TRIAL NAME/ IDENTIFIER	PHASE		SETTING/ LINE OF TREATMENT	INTERVENTIONS	ENDPOINT
***NEOADJUVANT prior to surgery***							
**Pembrolizumab**		NCT02707666	I		Neoadjuvant	Pembrolizumab IV every 21 days for 3 cycles Pemetrexed/cisplatin IV after surgery every 21 days for 4 cycles	- IFNγ gene expression profile response rate - Measurement of events
**Pembrolizumab**		NCT02959463	I		Adjuvant to radiotherapy	Pembrolizumab IV over about 30 min on Day 1; courses repeated every 3 weeks for up to 2 years Hemithoracic radiation therapy	- Safety and tolerability of pembrolizumab after radiation therapy - PFS, OS
**Durvalumab plus Tremelimumab**		NCT02592551	II		Neoadjuvant	Durvalumab IV 1500 mg once Tremelimumab IV 75 mg once	- Intratumoral ratio of CD8 T cells/Tregs - Percentage of ICOS and CD4 T cells - Quantitative assessment of PD-L1
**Atezolizumab**		NCT03228537	I		Neoadjuvant	Atezolizumab IV over 30-60 min Pemetrexed disodium IV over 10 min Cisplatin IV over 2 hours on Day 1. Cycle repeats every 21 days for 4 cycles	- Feasibility and safety of neoadjuvant treatment - PFS, OS
**Nivolumab +/- Ipilimumab**		NCT03918252	II/III		Neoadjuvant	Nivolumab IV 3 mg/kg on Days -42, 28, And 14 Ipilimumab IV 1 mg/kg on Day - 42 prior to surgery	- Feasibility and safety of neoadjuvant treatment - Pathological and radiographic response - Assessment of toxicity
***FIRST-LINE TREATMENT***							
**Nivolumab plus Ipilimumab** ***(191) (192)***		NCT02899299	III		First-line	Nivolumab IV 3 mg/kg once every 2 weeks Ipilimumab IV 1 mg/kg once every 6 weeks	- OS, ORR, DCR PFS - OS, ORR, PFS according to PD-L1 expression
**Atezolizumab**		NCT03762018	III		First-line	Atezolizumab IV 1200 mg on Day 1 every 3 weeks Bevacizumab IV 15 mg/kg on Day 1 every 3 weeks Carboplatin IV 4-6 cycles with AUC 5 Pemetrexed IV 500 mg/m^2^ on Day 1 every 2 weeks	- OS, PFS, ORR, TTF, DoR - Assessment of adverse effects
**Durvalumab**		NCT02899195	II		First-line	Durvalumab IV 1120 mg over 60 min, before chemotherapy Pemetrexed IV 500 mg/m^2^ over 10 min Cisplatin IV 75 mg/m^2^ over 2 hours	- OS, PFS, TTP, ORR - Assessment of adverse effects
**Pembrolizumab**		NCT02784171	II		First-line	Pembrolizumab IV 200 mg over 30 min on Day 1, every 21 days up to 2 years	- PFS, OS
***SECOND-/THIRD-LINE TREATMENT (single agent administration)***							
**Tremelimumab**	NCT01843374			II	Second- or third-line	Tremelimumab IV 10 mg/kg every 4 weeks for 7 doses	- OS (3 yrs) - OS (18 months), PFS, ORR
**Pembrolizumab**	NCT02054806			IB	Beyond front-line	Pembrolizumab IV 10 mg/kg on Day 1 of every 2-week cycle for up to 2 years	- Best overall response using RECIST - PFS, OS, DOR
**Nivolumab**	NCT02497508			II	Second-line	Nivolumab IV 3 mg/kg, administered every two weeks	- DCR - OS, PFS, TTP, ORR, safety, and tolerability
**Nivolumab**	NCT03063450			III	Relapsed	Nivolumab IV 240 mg over 30 min on Day 1 every 14-day cycle	- OS, PFS ORR, quality of life, toxicity, and cost effectiveness

#### Neoadjuvant Therapy

Neoadjuvant therapy is a novel strategy with the goal of “preparing” the tumor for consequent resection. It is not possible to remove the microscopic disease residue with surgery alone, whereas with ICIs, the antitumor immunity towards cancer antigens can be enhanced, favoring elimination and thus preventing postsurgical relapse (62). The ability to remove the spread of tumor cells is due to the immune system activation triggered by ICIs, which allows effector immune cells to circulate within the bloodstream and reach sites far from the primary tumor. This is the major difference from chemotherapy performed prior to surgery, which can only reduce the tumor mass (62). Indeed, the effectiveness of chemotherapy as adjuvant treatment is still an open debate; a recent study highlighted an equivalent risk of death for patients receiving neoadjuvant or adjuvant chemotherapy, thus suggesting no significant difference between the two regimens (63). Accordingly, immunotherapy appears to be a reasonable alternative to chemotherapy and several clinical trials are undergoing to prove the effectiveness of neoadjuvant treatment, some of which are discussed below. Pembrolizumab has been investigated due to its role in blocking PD-1/PD-L1 engagement, allowing increased T-cell activation against cancer cells (64). Garon et al. showed that, when at least 50% of tumor cells express PD-L1, there is a correlation with longer progression-free and overall survival in patients with non-small-cell lung carcinoma treated with pembrolizumab (65). However, taken alone, this marker is not sufficient to provide exhaustive information regarding possible clinical outcomes (65). An interesting study on a melanoma phenotype characterized by high PD-L1 expression on cancer cells and CD8 T cells with an IFN-γ gene expression profile proved the utility of these two markers in the prediction of possible clinical outcomes after ICI therapy (66). Considering that about one-third of MPM patients carry these features, it is reasonable to identify the IFN-γ gene expression profile before and after ICI treatment on tissues harvested from enrolled patients (66). This was exactly the primary scope of a window-of-opportunity pilot trial (NCT02707666) that aimed to evaluate the immune-related effects of pembrolizumab in the tumor microenvironment when exploited as a neoadjuvant therapy. Specifically, 15 MPM patients with resectable tumors were subjected to cycles of pembrolizumab injection, followed by surgical resection (at least 4 weeks after the third dose) and then adjuvant chemotherapy with cisplatin and pemetrexed (4 cycles). The completion of this clinical trial is planned for 2025; nevertheless, the obtained findings could provide meaningful insights concerning the discovery of prognostic markers that can predict responses to immunotherapy and the overall effectiveness of this combinatorial approach. Another interventional phase I study (NCT02959463) aims to assess biomarkers of interest, including cytokines, measurements of T-cell activation, and serum exosome micro-ribonucleic acid (RNA) following the delivery of pembrolizumab after radiation therapy to treat MPM. The rationale behind this approach involves recent findings on the immunomodulatory activity of radiation therapy. In fact, after local treatment, the upregulation of TILs and the activation of class I MHC has been shown, allowing the reaction of the immune system against tumor neoantigens (67). Therefore, the combination of radiotherapy with pembrolizumab administration might be able to enhance the antitumor effects (67). Investigations in the clinic are still ongoing for 24 participants subdivided into two cohorts, one being treated with hemithoracic radiation and consequent pembrolizumab administration and the other receiving palliative radiotherapy and ICI treatment according to a defined schedule. Another interventional study analyzed the effects of treatment with durvalumab and tremelimumab before surgery (NCT02592551). These two ICIs work differently from pembrolizumab because durvalumab binds PD-L1 to cancer cells, while tremelimumab is an anti-CTLA-4 agent that acts against T cells (67). Here, the effectiveness of this neoadjuvant treatment was detected through the quantification of different parameters that are mainly correlated with the intratumoral infiltration of immune cells. In fact, it is well-established that immune-inflamed tumors are characterized by greater CD8+ T-cell infiltration and higher PD-L1 expression, leading to a better response to immunotherapy (68). However, other types of immune cells (Tregs) are attracted to the tumor microenvironment, where they exert their modulatory function of blocking the activity of effector T cells. A high infiltration of Tregs results in a poor prognosis and survival rate (69). Furthermore, CD4 T cells participate in the antitumor immune response by secreting cytokines and stimulating the recruitment of effector cells. Due to this role, their presence within the tumor is fundamental (70). Therefore, the intratumoral CD8+/Treg ratio was determined, a quantitative assessment of PD-L1 was conducted, and the percentage of infiltrating CD4 T cells was detected in this clinical study to further identify the most suitable predictive markers for this kind of combination therapy. However, the trial is still ongoing, and results are not available yet.

Additionally, nivolumab and ipilimumab are being investigated as neoadjuvant agents in an interventional study with 30 participants enrolled (NCT03918252). The primary endpoints are the treatment’s feasibility and safety as well as the pathological (defined as ≤10% residual viable tumor cells in the resection specimen) and radiographic responses. By 2026, it should be possible to assess the effectiveness of this method, and it may be possible to exploit the great potential of these drugs. By contrast, the phase I pilot study NCT032285537 evaluated another option to pre-treat MPM in stage I-III with Pemetrexed and Cisplatin combined with Atezolizumab, which is then exploited as maintenance treatment; this latter can block the immune system activation through the PD-L1 binding on cancer cells surface. The theory behind this combinatory therapy prior to surgery is the reduction of tumor size to help with the surgical removal and the elimination of spread micro-metastases (62). However, preliminary results have been recently presented and they reduced the initial excitement. Specifically, 7 out of 21 enrolled patients, they did not complete the neoadjuvant therapy due to disease progression (n=4), toxicity (n=2) and death for sepsis correlated to grade 4 non-immune related renal and respiratory failure (n=1). Furthermore, at the time of the analysis, median OS has not been achieved and median PFS was 18.6 months (71). Despite the high expectations of this innovative approach based on immune checkpoint inhibitors in the preoperative condition to gain better performances, huge efforts and investigations are strongly required in clinical trials to identify benefits from this challenging strategy.

#### Immune Checkpoint Inhibitors as a First-Line Treatment

ICIs have been proposed as a first-line treatment in addition to the available standard of care (**Table 1**). The results of a relevant clinical trial, CheckMate743 (NCT02899299), have permitted the FDA approval of the combined treatment nivolumab plus ipilimumab for nonresectable MPM patients (72). This study enrolled 605 patients, and some of them were randomly treated with immunotherapeutic drugs, while the others received pemetrexed and cisplatin (73). Findings from this large, randomized study suggested a clinically meaningful improvement in terms of overall survival with the use of immunotherapy compared with the use of platinum and pemetrexed-based chemotherapy (41% vs. 27%) (73). Moreover, the prevalence of side effects was higher after immunotherapy, although these were manageable and could be treated with steroids or other supportive approaches (73). Nevertheless, several benefits were highlighted, particularly across histological subtypes. For instance, the median overall survival after treatment with nivolumab plus ipilimumab was consistent between epithelioid histology tumors (18.7 months) and nonepithelioid tumors (18.1 months) (73). Nevertheless, a recent comparative effectiveness study was conducted to compare the 3 main clinical trials of first line setting for MPM: the SoC cisplatin and pemetrexed (MPS), nivolumab and ipilimumab (CM73) and bevacizumab combined with the SoC (MAPS) (74). Through an accurate reconstruction of Kaplan-Meier curves, no significant improvements in terms of overall survival was detected for one treatment over the other. Moreover, potential biases on censored patients have been detected, leading to hesitations that should be further investigated and clarified to strengthen the reliability of the study (74). As well as that, another study aimed at comparing individual patient data reconstructed retrospectively from 4 clinical trials: the same considered in the other study and durvalumab combined with SoC. They showed comparable efficacy to the standard of care pemetrexed and cisplatin, providing slight significant survival benefits (59). Practically, apart from these retrospect analyses, how clinicians are applying this regimen in the real-world circumstances is still unclear, mainly in patients excluded from the trial because of autoimmune diseases, previous history of tumors, hampered laboratory parameters ecc. Therefore, the effectiveness and safety of the novel treatment needs to be further validated, mainly to find out the best regimen for aforementioned patients in daily clinical practice (75). Other clinical studies are currently ongoing. Among them, the BEAT-Meso study (NCT03762018) is evaluating the use of atezolizumab in combination with bevacizumab and chemotherapy in a front-line setting with the final aim of assessing the overall and progression-free survival, since bevacizumab is an antiangiogenic drug that has recently been demonstrated to be effective for the management of the disease (6). In addition, another open label, phase II study (NCT02899195), DREAM, is using a combination therapy consisting of durvalumab with pemetrexed and cisplatin as a first-line treatment. This procedure was approved for treating small-cell lung cancer, thus suggesting its possible exploitation for mesothelioma patients (76). Early available results have shown that 57% of the participants remained alive with progression-free survival after 6 months, thus demonstrating the higher benefits of the treatment compared with chemotherapy alone, while partial responses were shown in 31% of the enrolled patients with a tumor reduction of more than 50% (31). Thus, so far, the DREAM trial has demonstrated that chemoimmunotherapy is a safe and effective strategy, opening the way for further randomized phase III studies (31). Considering the potential of combined treatment using ICIs and chemotherapy, another study is currently ongoing to evaluate the employment of pembrolizumab, pemetrexed, and cisplatin in both single and combination treatments (NCT02784171). This approach is crucial to further clarify whether immunotherapy is more effective as a monotherapy or in association with the current standards of care. Specifically, 502 participants were recruited and then subdivided into three groups that were subjected to three different treatments to measure the progression-free and overall survival and assess the number and severity of adverse effects. The future results of this clinical trial may help to determine which type of therapy could provide better outcomes.

#### Immune Checkpoint Inhibitors as Second- And Third-Line Treatments

ICIs may also be useful as second- or third-line treatments when all previous strategies have failed. The use of an anti-CTLA-4 antibody called tremelimumab was investigated in the DETERMINE study (NCT01843374). This study involved 569 patients who had previously received other treatments (77). The obtained results suggest that tremelimumab did not significantly increase the overall survival rate at 18 months (17.4%) with respect to a placebo (18.2%) (77). Moreover, no clinically meaningful differences in progression-free survival were detected between the two investigated groups. In addition, the safety profile was shown to be adequate, thus suggesting its possible synergistic combination with other drug agents (77).Better findings were obtained in the KEYNOTE-028 study (NCT02054806), which investigated the use of the anti-PD-1 antibody pembrolizumab as a second-line treatment for advanced biomarker-positive solid tumors, including PD-L1-positive tumors in mesothelioma patients (78). Every patient was treated with a defined antibody dose for up to 24 months, and the collected evidence showed that 20% of patients had a partial response, while 52% achieved a stable disease state (78). Some treatment-related adverse effects were also observed, such as fatigue, nausea, and arthralgia, while a limited number of patients experienced more severe events. Nevertheless, no treatment-related mortalities were noted (78). In conclusion, pembrolizumab seems to be well-tolerated and can induce antitumor immunity for patients with PD-L1-positive tumors; however, the results regarding efficacy and response durability need to be confirmed with other, more specific studies (78). Unlike the aforementioned clinical trial, the NivoMes study (NCT02497508) involved the use of the anti-PD-1 antibody nivolumab as a monotherapy in patients with progressive mesothelioma without considering the PD-L1-positive selection criterion (79). Nivolumab is still being widely investigated because of its encouraging effects. In fact, another study exploited it to treat patients with relapsed malignant mesothelioma (NCT03063450) (80). It is known as CONFIRM and it represents the first placebo-controlled, phase III trial of a PD-1 agent in mesothelioma (80). Notwithstanding, the improvements regarding the overall survival in treated groups are still modest; indeed, with the nivolumab regimen the overall survival was 10·2 months, while the placebo group 6·9 months (81). On balance, although there are no supporting evidences to demonstrate immunotherapy as better intervention than chemotherapy in second-line settings, this randomized trials have provided meaningful data, in the short period, that clinicians and patients can exploit for treatment purposes (82).

### Use of Preclinical Models for Malignant Mesothelioma

Animal models of malignant mesothelioma are utilized to assess disease pathogenesis and to generate accurate preclinical models for identification of new treatment modalities that might move forward in clinical development (83). Unfortunately, all the desirable features will be unlikely found in a single animal model, but the cancer model should mimic important features of human mesothelioma, such as its pathology, gene expression profile, the inflammatory phenotype (84). Animal models can capture some of the complexity of the *in vivo* tumour environment that is known to contribute to disease progression and drug responsiveness. One major issue of animal models is the time and cost. Up to now animal models are crucial for drug testing and have significantly contributed to our understanding of malignant mesothelioma. The most important model include genetically modified mice, asbestos-induced murine models as well as patient-derived xenografts (PDX) models.

Genetically modified models have been generated through the changes of genes known to be involved in human mesothelioma pathogenesis. Mice with mesothelial-specific deletion of Nf2, p53 and Ink4a/Arf were generated using intrathoracic injection of Adeno-Cre virus in homozygous and heterozygous conditional knockout (CKO) mice of Nf2;p53, Nf2;Ink4a/Arf and Nf2;p53 carrying an inactive Ink4a allele (Nf2;p53;Ink4a*) (83, 85). However, in humans with malignant mesothelioma, it is the epithelioid histological subtype that predominates, while most CKO mice developed sarcomatoid mesothelioma. MM can also be developed through murine exposure to asbestos fibres. The most asbestos-induced murine models have been developed by intraperitoneal injection of asbestos (86–88). Several studies showed that inactivation of *Bap1, Nf2, Ink4a/Arf* and *Tp53* resulted in higher incidence and faster progression of MM in comparison to the wild-type mice treated with asbestos (83, 89).The long latency of this model is both its strength and its main weakness. On one hand, it gives an important tool to investigate molecular events that occur during the latency period, but the time required to develop tumours (up to 2 years), make it difficult for drug testing (83). To study immunotherapies for mesothelioma, syngeneic murine models can be considered (90–92). In these models, established murine cell lines are injected into the immunocompetent host, for example, subcutaneous or intraperitoneal injection of murine mesothelioma cells: AB1, AB12, AB22 cells in BALB/c mice or AE17 cells in C57BL/6J mice and F4-T2 cells in F344 Fischer rats (83, 93–95). Both peritoneal and pleural mesothelioma share similar biology to most available human MM lines, but murine mesothelioma cell lines suffer from having undergone clonal selection resulting in adaptation to *in vitro* culture. Finally, murine syngeneic tumour models fail to mimic the tumour heterogeneity seen in the clinic (83). Finally, subcutaneous or intrathoracic injection of human MM cell lines into immunodeficient mice are frequently used to study mesothelioma biology and treatment strategies (96–98). Immortalized and highly passaged human mesothelioma cell lines lack many characteristics of their original tumours. Although PDX models are a useful tool to study the response of patients to specific treatments, the lack of an immune system reduces their utility for testing immunotherapies (83, 96).

## Future Prospects in Mesothelioma Therapy

The current available standards of care are not significantly resolutive to allow the complete recovery of affected patients; therefore, new treatment strategies are in high demand. In the following sections, we provide novel insights regarding mesothelioma therapy.

### Oncolytic Viruses

Another emerging treatment, which has great potential for use in mesothelioma patients, is oncolytic virotherapy (**Table 2**). This is based on the ability of genetically modified viruses to replicate and kill cancer cells as well as boost the immune system’s activation towards the tumor microenvironment (6, 99–103). ONCOS-102 is an oncolytic adenovirus with a chimeric serotype 5/3 that is able to enhance interactions with the corresponding adenovirus receptors. It carries a deletion of 24 base pairs in the E1A region, allowing selective replication within cancer cells (99). It is the only oncolytic adenovirus that has been investigated in the clinic as a possible treatment option for MPM (104). Moreover, the ONCOS-102 genome is armed with a transgene encoding GM-CSF, which can induce immunostimulatory effects (105–107). Its safety and immunological activity were demonstrated in the randomized phase I clinical study NCT02879669, in which the intrapleural administration of ONCOS-102 combined with the injection of the current standards of care (pemetrexed and cisplatin) was compared to the administration of the latter as controls. Interestingly, an increase in the intratumoral concentration of cytotoxic T cells was detected in the experimental group (10 patients out of 15) with respect to the control group. In addition, the treatment induced a polarization from M2 to M1 macrophages, indicating immune stimulation (104). Moreover, the expression of PD-L1 was reported to be upregulated in 9 patients out of 15 in the group treated with the virus, thus supporting the possibility of combining oncolytic viral therapy with immune checkpoint inhibitors to obtain a synergistic effect (104).

**Table 2:** Clinical trials employing oncolytic viruses.

ONCOLYTIC VIRUS		TRIAL NAME/IDENTIFIER	PHASE	SETTING/LINE OF TREATMENT		INTERVENTIONS	ENDPOINT
***Adenovirus***							
**ONCOS-102**		NCT02879669	IB/II	First-line		ONCOS-102 Intrapleural Cycle 1: on Days 1, 4, 8, 36 Cycle 2: on Day 78 Cycle 3: on Day 120 Pemetrexed and cisplatin IV in 21-day cycles starting on Day 22	- Safety and tolerability profile - Number of patients with induction of tumor-specific CD8 T cells in PBMC - OS, PFS
***Measles virus***							
**MV-NIS**		NCT01503177	I	First-line		MV-VIS Intrapleural On Day 1 and every 28 days up to 6 courses	- Maximum dose tolerated - Adverse event profile - Safety profile
***Vaccinia virus***							
**GL-ONC1**	NCT01766739		I		First-line	GL-ONC1 intrapleural as a single dose and then escalating to three consecutive daily doses	- Maximum tolerated dose - Safety and tolerability
***Herpes Simplex virus***							
**HSV1716**	NCT01721018		I/II		First-line	HSV1716 intrapleural	- Safety and tolerability - Evidence of viral replication and cancer cell death

Another trial aiming to determine the maximum tolerated viral dose and to monitor its distribution within the tumor is currently ongoing. This trial is using a vaccinia oncolytic virus, GL-ONC1 (NCT01766739), which is a double-stranded DNA virus bearing cytolytic capacity for a wide range of tumor types (108). It is considered a good candidate for clinical studies due to its well-established safety profile (108). GL-ONC1 contains three transgenes encoding Ruc-GFP, β-glucuronidase, and β-galactosidase. It is able to enhance tumor specificity as well as permit the real-time monitoring of tumor cell infection (108). This dose-escalating study enrolled 18 participants who were subjected to an initial intrapleural single dose of GL-ONC1, followed by escalation to three consecutive daily doses in those with malignant pleural effusions. Once completed, the findings may provide extremely useful information for the treatment of the most severe type of disease in patients with pleural effusions.

Next interesting study focused on the use of a conditionally replication-competent virus developed through the deletion of the ICP34.5 gene (109). PART A of the study was an open-label, single-center trial (NCT01721018) with the aim of assessing the safety and feasibility of the treatment. The study first enrolled patients with unresectable MPM who only received a single dose of the virus through an indwelling catheter into the pleural space. PART B, instead, was an open-label study in which patients received two or four single doses of the virus at weekly intervals in order to determine the maximum tolerated dose. Regarding the safety profile, HSV1716 was demonstrated to be well-tolerated despite the occurrence of some adverse events, such as fatigue/lethargy, pyrexia, and influenza-like symptoms. Overall, the level of tolerability was acceptable for both single and repeated-dose treatments (109). Moreover, the level of effectiveness in terms of the Th1 antitumor response was determined to be robust and encouraging. In fact, the levels of IFN-γ, IL-2, and TNF-α in the pleural fluid increased after viral administration in 8 patients out of 11, thus suggesting a direct correlation with the induction of meaningful antitumor immunity (109). Oncolytic virotherapy is a rapidly growing field of immunotherapy that has been studied across a broad spectrum of malignancies (103, 110–113). Mesothelioma is a good candidate for studying oncolyses, given its frequently localized pattern of growth and location. Therefore, despite it being a relatively rare carcinoma, the multitude of viral studies conducted on mesothelioma have generated insights that can be applied to other cancer types. Importantly, cancer virotherapy is an attractive alternative to conventional treatments because it shows a wide range of antitumor effects, in addition to its ability to induce an antitumor immune response (114).

### Combination of Immune Checkpoint Inhibitors and Oncolytic Viruses

Many preclinical and clinical studies have considered the combination of ICIs and oncolytic viruses as a suitable approach for treating different malignancies. An open-label, multicenter, phase IB trial (NCT01740297) investigated the combination of T-VEC, an oncolytic virus approved for the treatment of advanced melanoma, and ipilimumab in patients with advanced melanoma in order to determine the incidence of dose-limiting toxicity and the objective response rate (115). The obtained results showed greater efficacy for the combinatory therapy compared to treatment with T-VEC or ipilimumab alone. However, this study only enrolled 19 participants, and its findings need to be confirmed in the ongoing randomized phase II trial (115).

The Hemminki group assessed the impact of local treatment with an adenovirus coding for TNF-α and IL-2 (TILT-123) on the systemic antitumor response in animals receiving anti-PD-1 therapy. In animals, the researchers investigated the tumor response to combined therapy with anti-PD-1. The virus was administered intratumorally, and CPI was administered systemically. The most efficacious treatment modality was found to be the combination therapy, which resulted in an abscopal effect. Moreover, the adenovirus therapy was associated with the formation of immunological memory. The tested virus was also reported to be effective for preventing the development of metastases. The researchers concluded that local treatment with TILT-123 improved the systemic response to anti-PD-1 therapy by remodulating the tumor microenvironment (116).

Similar observations were also shown by Ylosmaki et al., who developed a novel oncolytic adenovirus encoding two immunostimulatory molecules: CD40L and OX40L (VALO-D102). The intertumoral administration of PeptiCRAd significantly enhanced tumor-specific T-cell responses, inhibited tumor growth, and activated systemic anti-cancer immunity in mouse models (B16.OVA and B16.F10.9/K1 melanomas). PeptiCRAd therapy, in combination with anti-PD-1 treatment, significantly enhanced tumor growth control in the tested groups (117).

Another intriguing oncolytic adenovirus that is currently being studied in many tumor types, including mesothelioma, is ONCOS-102. Preclinical investigations have provided evidence of a synergistic antitumor effect in humanized mice bearing melanoma tumors treated with ONCOS-102 in combination with pembrolizumab, which can be explained by the capacity of the virus to trigger an immunogenic cancer cell death that subsequently supports the activation of T cells by pembrolizumab by suppressing the PD-1/PD-L1 interaction (118, 119). This shows that this treatment could be beneficial for patients with melanoma, and it warrants further testing in the clinic. Promising data were reported in a phase 1/2 trial, where combination therapy with ONCOS-102 and SoC chemotherapy (pemetrexed/cisplatin) was administered as a first-line and later MPM treatment, and the safety, immune activation, and clinical efficacy were compared to those for treatment with SoC alone (NCT02879669). A total of 31 patients were enrolled in the trial, with 20 patients in the treatment group receiving ONCOS-102 plus SoC chemotherapy and 11 patients in the control group receiving SoC only. Thirty-month follow-up testing has now been completed. At the 30-month follow-up point, the mOS was 25.0 months for the subgroup of randomized first-line ONCOS-102-treated patients (n=8). Immune activation was assessed in tumor biopsies taken before and after ONCOS-102 treatment. The tumor tissue analyses revealed ONCOS-102-induced modulation of the tumor microenvironment with an increased T-cell population. The reported immune activation was associated with tumor responses and was most pronounced in patients with better survival outcomes.

Despite the promising results with combination therapies, there are still some issues that have been encountered in these studies; for instance, the timing of immune checkpoint inhibitor administration after virotherapy is crucial for the efficacy of both strategies (120). Administering it too early could immediately enhance the innate and adaptive antiviral response, thereby reducing the virus’ spread and consequent oncolytic activity. By contrast, later injection could restrict the synergistic effect with the virus that is needed to clear malignant cells (120). Therefore, more extensive studies focusing on the further investigation of the mechanism of action and drug biology in combination with different treatment schemes should be conducted in order to enhance the efficacy of combination approaches.

### Cancer Vaccines

Cancer vaccines rely on leveraging specific functions of APCs to trigger higher T-helper cell responses and subsequently induce cytotoxic effector T-cells. Therapeutic vaccinations consist of either whole-cell vaccines or specific peptide antigen preparations able to augment the adaptive response by increasing antigen presentation (121). First of all, dendritic cells are increasingly used as vaccine adjuvants due to their peculiar ability of induce a CD8 T cell infiltration, which is well known to be correlated to higher overall survival in mesothelioma patients (58). Matured DCs upon cytokine cocktail exposure, are then exposed to autologous tumor cell lysate, a meaningful source of antigens (known as MesoPher vaccine); hence, they can induce a remarkable effector T cells activity against malignant clones, as demonstrated in mesothelioma-bearing mouse model. Encouraging preclinical results led researchers to move towards a first human trial involving 9 patients: 5 were receiving pulsed DCs after chemotherapy and 4 were treatment naïve. Primary outcomes highlighted safety and feasibility of the regimen, and no dose-limiting or major toxicities were observed; moreover, the dose of 25 million cells triggered radiographic responses, suggesting its relevance as optimal dose level (122). Since this study recruited a limited number of patients, consistent conclusions cannot be drawn, though it allowed the design of a randomized phase II/III trial (NCT03610360) to study the efficacy of DCs loaded with allogenic tumor lysate in MPM patients receiving first line chemotherapy. Primary endpoints will be overall and progression-free survival to potentially consider DCs as a new treatment option (123). The second class of cancer vaccine regards antigen-specific preparations, which are more easily produced in large scale and more reproducible than the aforementioned technique. In mesothelioma treatment field, Wilms’ tumor suppressor gene (WT1) has been studied as cancer vaccine due to its ability to induce CD4 and CD8 WT1-specific reactions (124). Interestingly, it was tested in a pilot trial in nine patients to preliminarily assess its safety and efficacy; afterwards, a randomized phase II trial (NCT01265433) has been planned to define the adjuvant activity of WT1 analogue vaccine after multimodal regimen. Cancer vaccine represents a promising strategy since preliminary results shed light on their safety and effectiveness, though further research and larger-scale clinical trials are strongly warranted to eventually consider their feasibility as frontline setting.

### Adoptive T Cell Therapy

A novel therapeutic opportunity is based on artificially enriching the amount of T lymphocytes capable of interacting with tumor antigens through the adoptive T-cell therapy (ACT). Generally, during tumor evolution, the T lymphocytes recognition of neoantigens could be defective and may hinder the consequent killing of targeted cells; therefore, improving effector immune cells activity is a conceivable way to favor the tumor removal (121, 125). Different strategies have been developed over the years to work on this therapy, but the most successful method was the designing of chimeric antigen receptors (CAR). They are recombinant receptors targeting tumor-associated antigens on cell surface independently from MHC presence, but mainly binding a wide range of targets as carbohydrates, lipids and proteins (126). Indeed, the most attractive antigen target in mesothelioma tumors is represented by mesothelin because of its overexpression and clear association with tumor aggressiveness (127). Mesothelin CARs are currently being studied in several phase I clinical trials supported by solid preliminary preclinical evaluations; as an example, mRNA electroporation technique was exploited to develop T cells with transient CAR expression and it induced strong antitumor response in xenograft models of human MPM (128). On the wave of these promising evidence, a phase I clinical trial (NCT01355965) was designed to clarify safety and feasibility of engineered T cells expressing mesothelin-targeting CAR. No off-tumor toxicity was detected after infusion, though meaningful clinical responses were not highlighted with this treatment setting (128). In addition, another trial (NCT02159716) was performed to evaluate a lentiviral transduction vector expressing anti-mesothelin second generation CAR; also, in this case adoptive T cells were well tolerated but the outcomes were not significantly improved. One of the major issues might be represented by the intravenous administration which can render the arrival of T cells to the tumor environment hardly possible, thus driving to less observed efficacy (127). Accordingly, another recent trial (NCT03054298) is investigating fully human derived anti-mesothelin CAR T cells intratumorally injected to bypass the spreading issu**e** (129). Data are not still available but altogether these trials may help to better design future clinical studies and provide novel immunotherapy-based approaches aimed at solving solid tumor malignancies.

### Novel Meso-Tailored Targets

**Figure 1:**
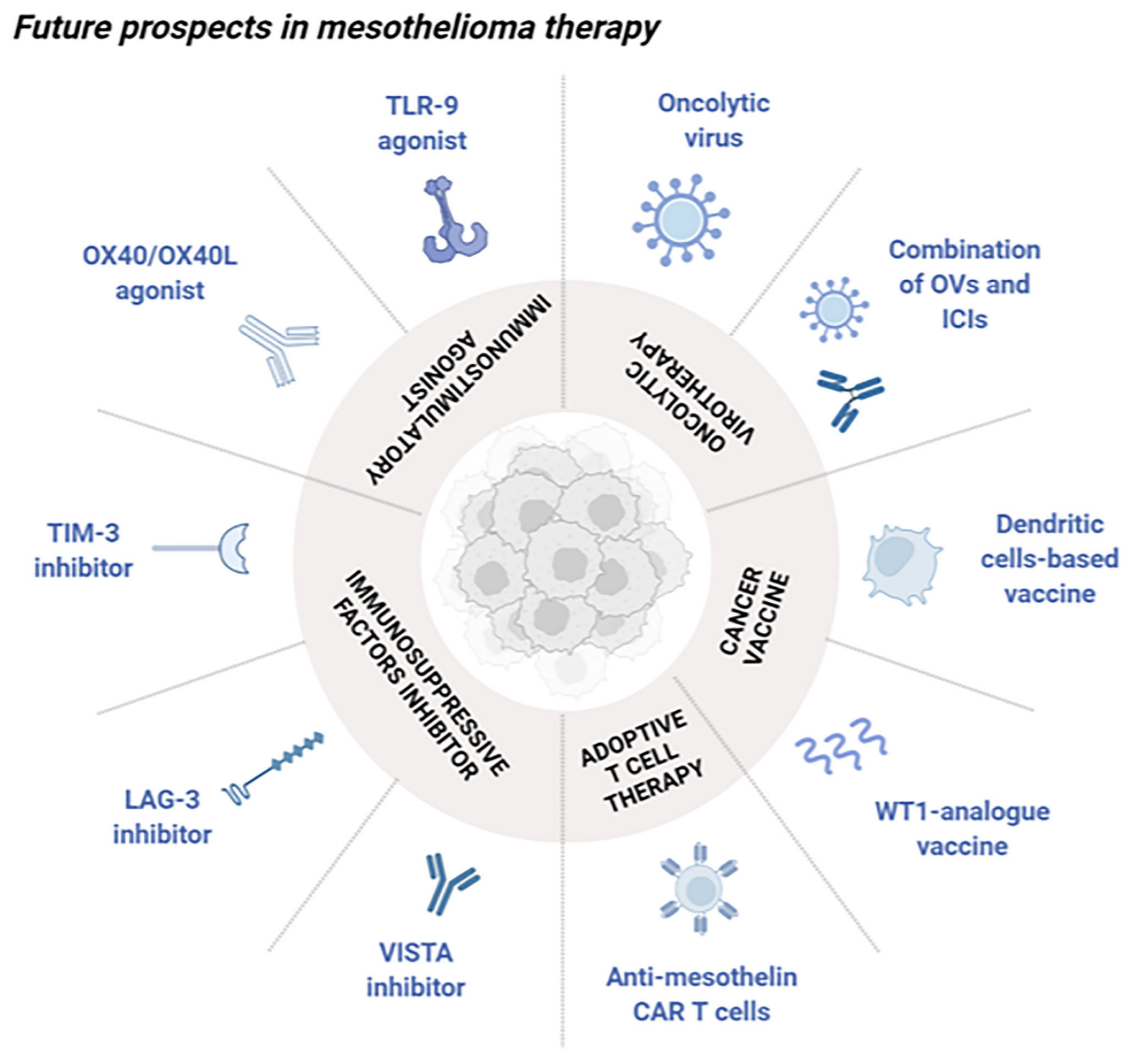
Schematic overview of future perspectives in mesothelioma immunotherapy. Novel approaches under investigation aimed at stimulating the immune system in the treatment of mesothelioma can be divided into different classes. Oncolytic virotherapy, alone or in combination with ICIs, can selectively kill cancer cells as well as boosting a strong antitumor immune reaction. Cancer vaccines are classified into cell-based vaccines with the purpose of activating effector T cells, and antigen peptides preparation (WT1-analogue vaccine) which selectively stimulates the immune system. Adoptive T cell therapy presenting anti-mesothelin CAR are mainly used for their ability to specifically recognize exposed tumor antigens. Immunosuppressive factors inhibitors such as VISTA, LAG-3 and TIM-3 inhibitors can block these molecules, known for their involvement into the tumor immune silencing. Immunostimulatory agents as OX40/OX40L and TLR9 agonists can boost a selectively immune reaction towards the tumor microenvironment. Created with BioRender.com.

Despite several immunotherapeutic approaches are under investigation in the current period, novel mesothelioma targets have been exploited to develop potential candidates for clinical use. Indeed, some of them are illustrated in the following sections, suggesting the importance of knowing tumor biology to rationally create innovative tailored strategies (**Figure 1**).

Immune checkpoints genes are widely investigated because of their involvement in the tumor immunosuppressive microenvironment, typical of solid cancers as mesothelioma. By mean of specific interactions with immune cells, they can block their activity, thus rendering the immune system unable to exert its protective function. Among the recent discovered genes, V-domain Ig suppressor of T cell activation (VISTA) exerts its activity by decreasing the production of cytokines by T cells and by reducing their proliferation (130) it has been shown to be highly expressed on the epithelioid subtype and an ongoing phase I clinical trial (NCT02812875) aimed at studying the efficacy of a small molecule inhibitor against VISTA in patients with advanced solid tumors or lymphomas (131). On top of that, lymphocyte activation gene-3 (LAG-3) is a receptor which can inhibit the activity and expansion of T cells, hence warranting immune homeostasis. Even though LAG-3 is not directly expressed by cancer cells, it is usually detected in pleural effusions of mesothelioma patients and tumor infiltrated lymphocytes in pleural effusions (132). Nowadays, inhibitors of LAG-3 are investigating in clinical trials to treat other types of malignancies like breast cancer; evidences appeared to be promising due to survival rate of 50%, which might provide consistent rationale to apply this strategy for mesothelioma treatment as well (131). Among markers responsible for the immunosuppressive milieu, T-cell immunoglobulin and mucin-containing protein 3 (TIM-3) is mainly expressed by immune cells (CD8 and CD4 T cells, macrophages, DC) and it induces suppression of Th1 response and enhances the activation of Tregs (133). It has been shown how TIM-3 is commonly found on PD-L1 positive mesothelioma tumors and the improved survival following anti-CTLA4 treatment is associated to a lower expression of TIM-3; as a result, the evaluation of its presence might be a reasonable predictive factor to foresee therapy-responding patients (134). Furthermore, different drugs targeting this peculiar marker are being investigated in clinical trials for the treatment of advanced solid tumors either alone or in combination with other immunotherapeutic strategies; however, results have not been published yet, and eventual benefits are still unknown (131). On the other hand, it could be intriguing to boost the antitumor immunity instead of reducing the immunosuppressive features. Therefore, interactions responsible for costimulatory signals in immune T cells are OX40/OX40L which belong to the TNF receptors superfamily and are involved in mesothelioma tumors (135). A preclinical evaluation evidenced the high expression of OX40 and CTLA-4 in tumor infiltrating T cells, driving to the rationale of targeting it to provide an effective immune response against the tumor progression. Remarkably, a synergistic activity was highlighted with anti-CTLA-4 agents thus triggering an increase in complete tumor regression from 20 to 80% (136). Likewise, Toll-like receptor 9 (TLR-9) describes a proper alternative target to obtain a meaningful immune activation into the tumor site. It is an intracellular DNA receptor, activated by DNA recognition and consequently able to induce a cascade which promotes transcription factors activation like NF-kB and AP-1 (Activator protein 1). As well as that, it can boost the acquired immunity through the release of cytokines and DC maturation. This is why, TLR-9 agonists might be reasonable candidates aimed at stimulating an antitumor response against the malignancy; as an example, CpG oligodeoxynucleotides injection showed strong activity on established AB1 mesothelioma model leading to long-term survival. Collected preclinical data sustained the use of immunostimulatory agents as novel treatment opportunities for mesothelioma tumors.

## Conclusions

Despite huge efforts to improve the understanding and treatment of MPM, clinical practice has not changed dramatically in recent decades. To accelerate the development of novel treatment options, rational and well-designed investigations should be performed, and personalized approaches should be investigated. Recently, the use of ICIs showed impressive clinical responses in patients with other solid malignancies. However, their impact on survival in MPM patients as single agents is small. Therefore, hope for patients with MPM comes from innovative therapies, such as oncolytic virotherapy in combination with ICIs and from the study of novel meso-tailored targets that might provide appealing opportunities to treat this severe malignancy.

## Author Contributions

LK and MG prepared the manuscript. GR prepared part of the manuscript. MS, KP, MW, SS and PC helped with the review and made modifications along with suggestions to improve the content. All authors contributed to manuscript revision, read, and approved the submitted version.

## Funding

LK was supported by the National Science Centre, Poland SONATINA (2019/32/C/NZ7/00156) and the National Institute of Public Health NIH – National Research Institute, Poland (1BWBW/2021, BW-3/2022). M.G. acknowledges PRID-J (Grant Number: GARO_SID19_02) funded by University of Padua. MS was supported by Centre for Advanced Materials and Technologies, WUT (Poland) and grant BioTechMed_LAB-1 IDUB (Excellence Initiative Research University).

## Conflict of Interest

Author LK is a shareholder in Targovax.

The remaining authors declare that the research was conducted in the absence of any commercial or financial relationships that could be construed as a potential conflict of interest.

## Publisher’s Note

All claims expressed in this article are solely those of the authors and do not necessarily represent those of their affiliated organizations, or those of the publisher, the editors and the reviewers. Any product that may be evaluated in this article, or claim that may be made by its manufacturer, is not guaranteed or endorsed by the publisher.
